# Population-based cohort study of the impacts of mild traumatic brain injury in adults four years post-injury

**DOI:** 10.1371/journal.pone.0191655

**Published:** 2018-01-31

**Authors:** Alice Theadom, Nicola Starkey, Suzanne Barker-Collo, Kelly Jones, Shanthi Ameratunga, Valery Feigin, on behalf of the BIONIC4you Research Group

**Affiliations:** 1 National Institute for Stroke and Applied Neuroscience, Auckland University of Technology, Auckland, New Zealand; 2 Department of Psychology, University of Waikato, Knighton Road, Waikato, New Zealand; 3 Department of Psychology, University of Auckland, Auckland, New Zealand; 4 School of Population Health, University of Auckland, Auckland, New Zealand; Uniformed Services University of the Health Sciences, Bethesda, UNITED STATES

## Abstract

There is increasing evidence that some people can experience persistent symptoms for up to a year following mild TBI. However, few longitudinal studies of mild TBI exist and the longer-term impact remains unclear. The purpose of this study is to determine if there are long-term effects of mild traumatic brain injury (TBI) four-years later. Adults (aged ≥16 years) identified as part of a TBI incidence study who experienced a mild-TBI four-years ago (N = 232) were compared to age-sex matched controls (N = 232). Sociodemographic variables, prior TBI and symptoms were assessed at the time of injury. Four years post-injury participants completed the Rivermead Post-Concussion Symptom Questionnaire, Hospital Anxiety and Depression Scale, Pittsburgh Sleep Quality Index and the Participation Assessment with Recombined Tools. Analysis of covariance was used to compare differences between TBI cases four years post-injury and controls, controlling for prior TBI and depression. A multiple regression model was used to identify the predictors of increased symptoms and reduced participation. The mild-TBI sample experienced significantly increased self-reported cognitive symptoms (F = 19.90, p = <0.01) four years post-injury than controls. There were no differences between the groups for somatic (F = 0.02, p = 0.89) or emotional symptoms (F = 0.31, p = 0.58). Additionally, the mild-TBI group reported significantly poorer community participation across all three domains: productivity (F = 199.07, p = <0.00), social relations (F = 13.93, p = <0.00) and getting out and about (F = 364.69, p = <0.00) compared to controls. A regression model accounting for 41% of the variance in cognitive symptoms in TBI cases revealed a history of TBI, receiving acute medical attention and baseline cognitive symptoms, sleep quality, anxiety and depression were predictive of outcome. The results indicate that whilst somatic and emotional symptoms resolve over time, cognitive symptoms can become persistent and that mild TBI can impact longer-term community participation. Early intervention is needed to reduce the longer-term impact of cognitive symptoms and facilitate participation.

## Introduction

Up to 95% of traumatic brain injuries (TBIs) are classified as being mild in severity.[1] Whilst the impact on cognitive, emotional and behavioural functioning of moderate and severe TBI have been well documented,[2] much less is known about the impact of mild-TBI, especially in the longer-term. Given 40% of mild-TBIs occur in early to mid-adulthood,[1] the societal and personal burden of mild-TBI may be higher than anticipated. For example, whilst the costs of moderate and severe injury are higher on a per person basis, as mild-TBI occurs more frequently than moderate to severe TBI, the overall costs of mild-TBI are three times higher.[3]

Whilst there is considerable variation in how TBI affects a person and their level of functioning,[4] people commonly experience a range of impairments referred to as post-concussion symptoms. These include cognitive (e.g., difficulty concentrating and taking longer to think), somatic (e.g., headaches, fatigue or dizziness) and emotional symptoms (e.g., feeling irritable, frustrated or restless).[5] Cognitive symptoms have been linked to reduced productivity at work,[6, 7] higher health service use, and engagement in antisocial behaviour.[8] Whilst many recover naturally from mild-TBI within weeks or months of the injury,[9, 10] evidence has now revealed that symptoms can persist for between 24% and 48% of those experiencing a mild-TBI one year post-injury.[9, 11–14] However, longitudinal, controlled, follow up studies within a mild-TBI cohort have not been conducted to determine whether symptoms persist in the longer-term and the impact of mild-TBI on community participation remains unclear.

One of the difficulties in studying mild-TBI is that some people do not attend hospital post-injury and seek assistance in the community from a General Practitioner or allied health professional such as a physiotherapist or choose not to seek medical attention at all. Additionally, mild-TBIs can often be overshadowed in the context of other injuries that are sustained concurrently which require more urgent medical attention. Consequently, studies that only explore mild-TBI cases that attend hospital services may not be representative of the mild-TBI spectrum. A further difficulty is that symptoms experienced following a mild-TBI can be caused by other medical conditions or illness.[14] Therefore, to determine the isolated effects of mild-TBI, it is important to compare the symptom experience to controls without history of mild-TBI. Additionally, whilst it is important to understand whether symptoms persist in the longer term, it is also vital to determine the impact that symptoms have on people’s ability to participate in everyday activities. In this study we compare the experience of post-concussion symptoms and community participation in a mild-TBI cohort, four-years post-injury, with age and gender matched controls to determine if there are long-term impacts following mild-TBI.

## Materials and methods

Ethical approval was obtained from the Northern Y Health and Disability Ethics Committee of New Zealand (NTY/09/09/095) and the Auckland University of Technology Ethics Committee (09/265).

### Participants

Participants who had experienced a mild-TBI were recruited through an TBI incidence and outcome study (*Brain Injury Incidence and Outcomes New Zealand (NZ) in the Community*, BIONIC) undertaken between 2010 and 2011.[15] The BIONIC study identified all TBI cases that occurred in the Hamilton and Waikato districts of NZ over a 12-month period. TBI was defined using the World Health Organisation criteria as an “acute brain injury resulting from mechanical energy to the head from external physical forces….the manifestations of TBI should not be due to drugs, alcohol, medications or caused by other injuries or medical conditions” (p115).[16] Participants were considered eligible if they had described an incident meeting likely to result in a TBI (as defined above) and met at least one of the following criteria; dazed or confused after the accident, loss of consciousness for 30 minutes or less, and/or not being able to remember what happened during the accident. As many people do not seek medical attention following injury, for self-reported cases details of the accident were obtained and reviewed by a diagnostic team of neurologists, clinicians and neuropsychologists to determine eligibility for the study. Severity of TBI was operationalised as a record of post-traumatic amnesia (PTA) for less than 24 hours and/or Glasgow Coma Scale (GCS) score of 13–15. Cases where there was no GCS or PTA recorded in the medical record were classified as being a mild-TBI.

**Fig 1:**
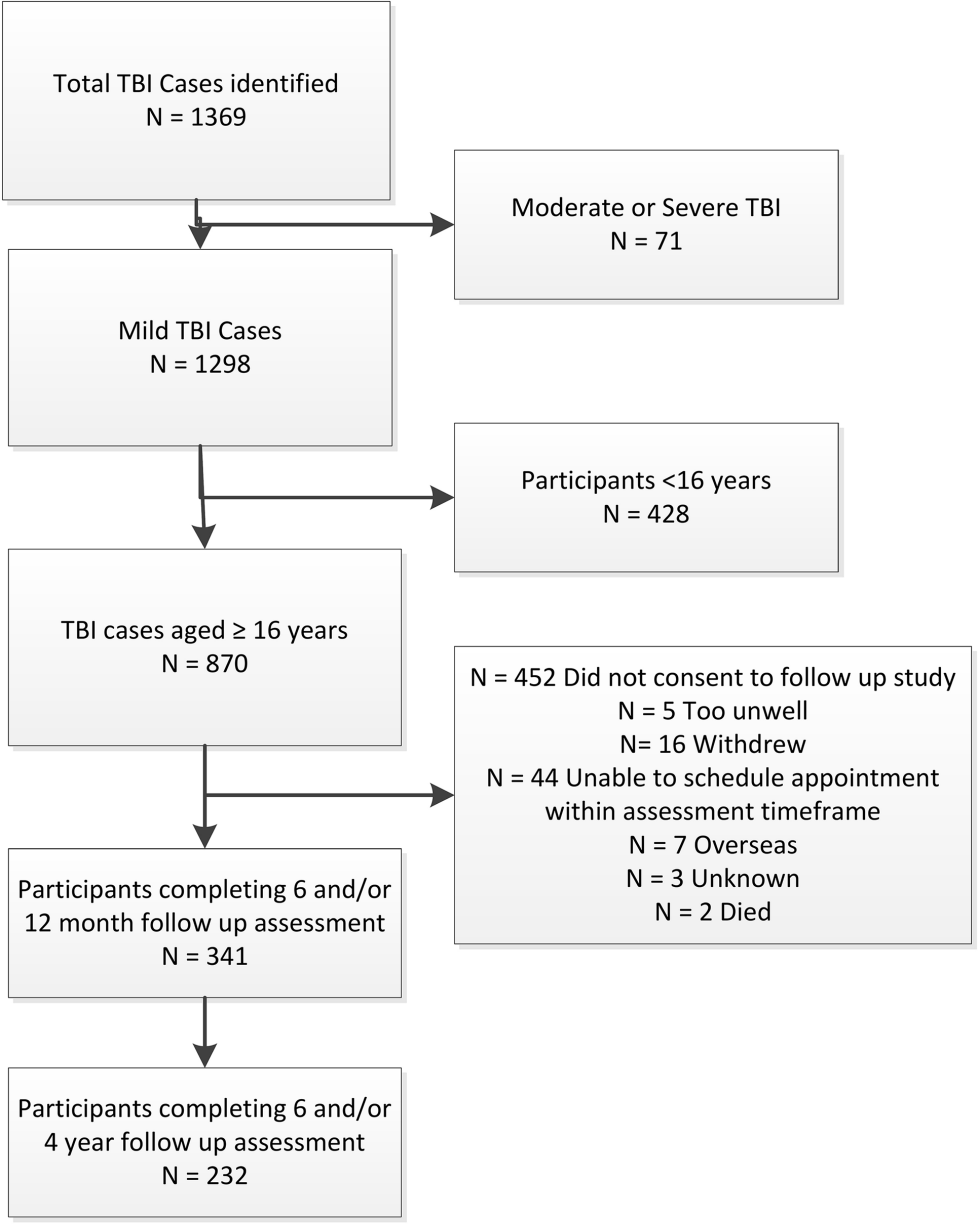
Participant flow diagram.

One strength of identifying cases through the incidence study, was that both cases presenting to hospital as well as cases receiving medical attention through community health care services, concussion clinics, sports clubs and self-referrals were identified. Participants were also identified through searches of claims made through NZ’s national accident compensation provider. This approach aimed to prevent potentially skewing any findings of longer-term impact through only including those who sought hospital based medical treatment following the injury. Whilst the incidence study included mild-TBI cases of all ages and severities, for the purposes of this study only those cases aged >12 years at the time of injury (thereby meeting the criteria of being aged ≥16 years at the four year follow up assessment to provide informed consent) and identified as having experienced a mild-TBI were extracted. From the incidence cohort of 341 adult participants who experienced a mild-TBI and agreed to complete a further follow up assessment 12-months post-injury, 232 (68.0%) completed a follow-up assessment at fouryears post-injury (see Fig 1).

Controls were initially recruited through general advertising in the community (email circulations, advertisements on electronic community noticeboards and posters in public spaces). Further participants were then targeted by age and sex through the Resarchnow registry (https://www.researchnow.com) to ensure each mild-TBI case could be age and sex matched to a control participant in NZ. As our mild-TBI cohort included people who sought medical treatment in the community or did not seek medical treatment at all post-injury, a community based rather than a hospital or medically based control group was deemed to be more comparable.

### Procedure

All cases identified as part of the TBI incidence cohort were contacted and invited to take part in an assessment four-years following their registering TBI injury. Participant’s ability to provide informed consent was determined by checking their level of understanding of the procedures, risks and benefits of taking part in the study. Assessments were completed in person or over the phone with a researcher or self-completed online based on participant preference. Flexibility in assessment administration was needed to enable as many people as possible to take part. The majority of mild-TBI case assessments (94.8%) were completed in-person with a researcher, with the remainder (3.8%) completed over the phone based on participant preference.

For both cases and controls socio-demographic information was recorded in addition to the TBIs sustained over the lifetime (prior to the TBI reported as part of the incidence study for TBI cases). Prior TBIs were determined by asking participants the number of occasions that they had previously experienced an incident where they hit their head and felt dazed or confused, lost consciousness or were able to remember what happened.

The Rivermead Post Concussion Symptoms Questionnaire[17] (RPQ) was designed to assess the frequency and severity of self-reported symptoms after a brain injury and was completed at baseline and four-years post-injury for TBI cases. The questionnaire includes a list of 16 cognitive, emotional and physical symptoms commonly experienced following a brain injury and that can also occur in everyday life. Participants are asked to indicate the severity they experience each symptom from 0 = Not experienced at all; 1 = No more of a problem; 2 = A mild problem; 3 = A moderate problem; to 4 = A severe problem. To enable the measure to be completed four-years post-injury and to be of relevance to those who have not experienced a brain injury participants, scores of 0 or 1 were combined and scored as (0) “not present”. A confirmatory factor analysis previously conducted in people following TBI revealed three factors identified as cognitive, emotional and somatic symptoms.[5] Item scores were summed to yield a score for each of these three factors as well as an overall total score to enable comparison with previous studies. TBI cases completed this assessment at baseline and four years post-injury and controls at one point in time.

Participation in everyday life was assessed four-years post-injury by the Participation Assessment of Recombined Tools (Part-O).[18] This scale consists of 17-items derived from three participation/community integration measures commonly used in TBI research. Each item asks about the person’s engagement in different activities in a typical week. Items are summed to yield three sub-scales assessing different domains of community participation including; productivity (e.g. In a typical week, how many hours do you spend in active homemaking, including cleaning, cooking and raising children?”), social relations (e.g. In a typical week, how many times do you give emotional support to other people, that is, listen to their problems or help them with their troubles?”) and getting out and about (e.g. In a typical month, how many times do you engage in sports or exercise outside your home?”). The measure has demonstrated construct, content and concurrent validity in people with TBI.[18] TBI cases completed this measure four years post-injury and controls at one time-point.

As depression and sleep quality have also been found to be predictive and a link between TBI and short to medium term outcomes following mild-TBI and were therefore assessed in order to account for their specific contributions to variance in outcome.

The Pittsburgh sleep quality index (PSQI) was used assess sleep quality four years post-injury. The PSQI[19] yields a global self-reported sleep quality index score based on responses to items exploring time taken to fall asleep, sleep duration, sleep efficiency, sleep disturbance, hypnotic medication use and daytime dysfunction. The PSQI distinguishes between good and poor sleepers and has demonstrated high sensitivity and specificity post-TBI.[20] TBI cases completed this measure four years post-injury and controls at one point in time.

The Hospital Anxiety and Depression Scale (HADS)[21] was administered four-years post-injury to explore the impact of mood on outcomes. The scale consists of two subscales, anxiety and depression, each based on 7 items. The HADS subscales have been found to be a reliable measure of emotional distress in TBI.[22] TBI cases completed this measure four years post-injury and controls at one point in time.

### Statistical analyses

Descriptive statistics were used to describe the participant samples. Chi square or t-tests were used to determine if there were any differences between the mild-TBI sample and controls on sociodemographic characteristics and contextual factors. ANCOVA was used to determine differences between the two groups on severity of symptoms and community participation. As prior TBI and depression have been found to be one of the most significant predictors of symptoms after mild-TBI, these were controlled for in the analysis of group differences.[9, 23] Cohen’s d effect size statistics were also calculated to show the magnitude of difference, with effect sizes of 0.19 considered as being trivial, 0.20–0.59 small, 0.60–1.19 moderate and >1.20 large.[24] A correlation matrix determined whether age, gender, ethnicity, living situation, medical consultation within 24 hours, comorbidities, prior TBI, depression, anxiety sleep quality and baseline symptom subscale scores and mode of assessment completion were significantly correlated with each outcome variable. Variables significantly associated with each outcome were entered into a multiple regression analysis to identify the predictors of outcome.

## Results

As shown in Table 1, TBI participants were identified from a range of sources. Of the 232 identified participants, 210 (90.5%) had sought medical attention post-injury, with 186 (88.6%) of those seeking medical attention within 24 hours. The majority of cases seeking medical attention attended hospital (70.2%), although the sample also included people seeking medical attention in the community (27.5%) as well as self-referrals who did not seek medical treatment (7.3%). There were no differences between the mild-TBI cohort and controls on sociodemographic and contextual variables, except for employment status (Table 1). There were significantly higher rates of post-injury unemployment in the mild-TBI group.

**Table 1:** Participant characteristics of the mild-TBI and controls samples.

	Mild TBI Sample at 4 years N = 232	Controls N = 232	Test of Difference(p-value)
**Mean age in years (SD)**	40.39 (18.5)	40.72 (17.0)	t = 0.196 p = 0.85
**Gender N (%)**			X2 = 0.00 p = 1.00
Male	130 (56.0)	130 (56.0)	
Female	102 (44.0)	102 (44.0)	
**Ethnic origin N (%)**			X2 = 0.537 p = 0.46
European	166 (71.6)	173 (74.6)	
Other	66 (28.4)	59 (25.4)	
**Living situation N (%)**			X2 = 4.85 p = 0.18
Living alone	32 (13.8)	29 (12.5)	
Living with others	35 (15.1)	23 (9.9)	
Living with family/spouse	164 (70.7)	176 (75.9)	
Unknown	1 (0.4)	4 (1.7)	
**Marital status N (%)**			X2 = 6.58 p = 0.08
Married/civil union/de facto	108 (46.6)	135 (58.2)	
Separated/divorced/widowed	32 (13.8)	23 (8.9)	
Never married or single	90 (38.8)	73 (31.5)	
Missing	2 (0.9)	1 (0.0)	
**Employment Status**			X2 = 5.51 p = 0.02
Full or part time employment	133 (57.6)	159 (68.5)	
Student	23 (10.0)	26 (11.2)	
Homemaker	9 (3.9)	4 (1.7)	
Retired	25 (10.8)	20 (8.6)	
Unemployed/on benefit	40 (17.3)	19 (8.2)	
Missing	1 (0.4)	0 (0)	
**Prior TBIs in lifetime N (%)**			X2 = 2.31 p = 0.31
None	104 (44.8)	90 (38.8)	
1 to 2	76 (32.8)	79 (34.1)	
3 or more	43 (18.5)	29 (12.5)	
Unknown	9 (3.9)	34 (14.7)	
**Cause of injury N (%)**			
Transport accident	56 (24.1)		
Fall	75 (32.3)		
Exposure to mechanical force	50 (21.6)		
Assault	46 (19.8)		
Other/unknown	5 (2.2)		
**Additional injury N (%)**			
Yes	157 (67.7)		
No	60 (25.9)		
Unknown	15 (6.5)		
**Source of identification**			
Hospital	151 (65.0)		
GP or accident clinic	34 (14.7)		
Self-referral	8 (3.0)		
Patient support organization	9 (4.0)		
National Compensation Claim	30 (12.9)		
**Medical attention sought within 24 hours N (%)**			
Yes	186 (80.2)		
No	46 (19.8)		

Of the 210 participants who sought medical attention after injury, 159 (75.7%) were discharged home, 9 (4.2%) received an onward referral, 6 (2.9%) left medical services before being seen by a clinician due to long wait times and 36 (17.1%) were hospitalised.

Comparing the mean scores on the outcome measures between the two groups, controlling for prior TBI and levels of depression (see Table 2), the mild-TBI group experienced significantly more self-reported cognitive symptoms four-years post-injury than controls. There were also differences between the groups in terms of participation four-years post-injury, with the mild-TBI group reporting significantly poorer participation across all domains compared to the controls. Effect sizes were most notable for productivity and being out and about, although the effects were small. A post-hoc power calculation revealed that the study had 90% power to detect an effect size of 0.2 (alpha = 0.05).

**Table 2:** Mean scores on the outcome measures between the two samples.

	Mild TBI Sample at 4 years	Controls	Test of Difference	Cohen’s D Effect size
	N = 232	N = 232	(p-value)	
	M (SD)	M (SD)		
**Post-concussion Symptoms (RPQ)**				
Total Score* (0–64)	16.54 (12.93)	17.00 (12.13)	F = 2.92, p = 0.09	0.02
Cognitive (0–12)*	4.24 (3.51)	3.53 b(3.45)	F = 19.90, p = 0.00	0.20
Somatic (0–36)*	8.21 (7.67)	8.88 (6.11)	F = 0.02, p = 0.89	0.10
Emotional (0–16)*	3.97 (3.84)	4.57 (4.06)	F = 0.31, p = 0.58	0.15
**Community Participation (Part-O)**				
Productivity (0–5)	2.84 (1.35)	3.57 (1.25)	F = 199.07 p = 0.00	0.27
Social relations (0–5)	2.44 (0.71)	2.55 (0.55)	F = 13.93, p = 0.00	0.05
Out and about (0–5)	1.90 (0.73)	2.36 (0.60)	F = 364.69, p = 0.00	0.33

* higher scores indicate poorer outcome

As shown in Table 3, the regression models all predicted more than 40% of the variance in long-term symptoms for mild-TBI cases. Baseline symptom scores were predictive of longer term cognitive and somatic symptoms with depression, sleep quality and prior TBI revealed as independent predictors of long-term symptoms in the models. Regression models explained less variance (3–27%) in participation outcomes, with only age and education independently predicting rate of productivity, and age and depression independently linked to getting out and about four-years post-injury. Only highest education level was correlated with social relations four years post-injury, and the overall regression model was not significant.

**Table 3:** Regression models of four year outcomes.

Four year outcome	Variables in model	Unstandardised Beta	t	p	95% CI	95% CI	F	SIg	R2
					Lower	Upper			
PCS	Constant	1.68	0.68	0.5	-3.26	6.61			
Cognitive symptoms	Medical attention	-0.82	-0.39	0.7	-4.99	3.56			
	Past TBI	-0.33	-0.67	0.51	-1.31	0.65			
	Sleep Quality	0	-0.01	0.99	-0.2	0.19			
	Baseline cognitive symptoms	0.36	3.45	0	0.15	0.58			
	Anxiety	0.16	1	0.28	-0.13	0.45			
	Depression	0.31	2.16	0.04	0.02	0.59			
							6.67	0.00	0.41
PCS	Constant	-1.24	-1.36	0.18	-3.06	0.59			
Emotional symptoms	Past TBI	0.82	1.98	0.05	-0.01	1.65			
	Sleep Quality	0.22	2.51	0.02	0.04	0.39			
	Baseline emotional symptoms	0.16	1.86	0.07	-0.01	0.33			
	Anxiety	0.16	1.17	0.25	-0.11	0.42			
	Depression	0.22	1.76	0.09	-0.03	0.47			
							11.51	0.00	0.50
PCS	Constant	0.15	0.04	0.97	-8.39	8.69			
Somatic symptoms	Medical attention	-2.53	-0.72	0.48	-9.59	4.53			
	Past TBI	2.17	2.48	0.02	0.42	3.92			
	Sleep Quality	0.33	1.89	0.06	-0.02	0.67			
	Baseline somatic symptoms	0.34	4.24	0	0.18	0.5			
	Anxiety	-0.06	-0.21	0.84	-0.58	0.48			
	Depression	0.01	-0.02	0.98	-0.5	0.51			
							8.47	0.00	0.47
PART-O	Constant	2.4	7.08	0	1.73	3.07			
Productivity	Age	-0.02	-5.79	0	-0.03	-0.02			
	Gender	0.25	1.79	0.08	-0.03	0.53			
	Education	0.23	2.87	0.01	0.07	0.39			
	Depression	-0.05	-1.99	0.05	-0.1	0			
	Sleep Quality	-0.02	-0.9	0.37	-0.06	0.02			
							11.71	0.00	0.27
PART-O	Constant	1.74	6.2	0	1.18	2.29			
Social relations	Education	0.18	1.8	0.08	-0.02	0.37			
							3.23	0.08	0.03
PART-O	Constant	2.11	16.62	0	1.86	2.36			
Out and about	Age	-0.01	-4.24	0	-0.01	0			
	Living alone	-0.06	-1.58	0.12	-0.14	0.02			
	Sleep Quality	-0.01	-0.48	0.63	-0.02	0.02			
	Anxiety	0	0.1	0.92	-0.02	0.02			
	Depression	-0.05	-3.74	0	-0.08	-0.02			
							12.07	0.00	0.24

## Discussion

This study has revealed initial evidence to suggest that people who have sustained a mild-TBI have significantly increased self-reported cognitive symptoms (including forgetfulness, poor concentration and taking longer to think) and reduced community participation four-years post-injury compared to controls. This difference remained significant even after controlling for prior TBI and depression, although the effect sizes were small. The persistence of cognitive symptoms four years post-injury, suggests that cognitive symptoms which fail to resolve in the acute phase post-injury are likely to become chronic, and impact participation without intervention. People experiencing acute cognitive symptoms, who have a history of TBI, sought acute medical attention, experience poor sleep quality and with high levels of anxiety or depression are more at risk of persistent longer term cognitive symptoms.

This is the first study to identify that adults experience significantly greater community participation difficulties than controls four years after their injury. These findings compliment previous research which has highlighted the impact of mild-TBI on work productivity four years after mild-TBI.[6, 25] Indeed, a changing sense of self, needing to manage limitations caused by the TBI symptoms and needing support to cope with the unpredictability of the future have been important factors affecting employment following mild-TBI. The current findings highlight that the impact of mild-TBI extends beyond paid employment and also affects wider community participation. Current interventions to increase participation have revealed limited impact and new approaches to increase participation after mild-TBI are needed.[26]

One of the strengths of this study was the inclusion of participants who attended hospital post-injury in addition to people who received medical attention in the community or whom did not seek medical treatment. Mild-TBI is a heterogenous group and the finding that presentation for medical advice post-injury was associated with increased somatic and cognitive symptoms is likely to reflect the injury severity, rather than indicating that medical advice is detrimental. The findings suggest that those seeking medical attention are those at greatest risk of poorer long term outcome and highlights this interaction as an important point of contact where interventions could help to improve long term outcome.

The significant differences observed between the mild-TBI cohort four-years post-injury and controls, whilst small, were notable given that it has been previously perceived that most cases of mild-TBI resolve in the days to weeks following injury.[27] This finding extends evidence of the identified effects of mild-TBI within a year post-injury.[4, 9, 14, 28, 29] by providing evidence of persistent effects four-years post-injury. It was interesting that there was less than a 0.1 change in the total RPQ mean symptom score identified in this study compared to the one year outcomes previously reported for this sample,[9] suggesting chronicity of symptoms. The findings in our community based sample are consistent with the findings of a recent systematic review of the impact of sport-related concussion, which revealed that some former athletes are at risk of cognitive impairment for many decades post-injury.[30] Exactly how long these symptoms persist for following mild-TBI remains unknown. Only six participants within this mild-TBI sample received active treatment following their injury and consequently it is hoped that the trajectory of symptoms could be improved with intervention and is an important area of exploration for future research. Indeed evidence of the effectiveness of early intervention and information provision has been encouraging, although the quality of evidence remains low and further trials of interventions specifically for mild-TBI are needed.[31]

The recovery journey following TBI has been found to be complex and influenced by a myriad of personal, social, and environmental factors.[32] It was interesting that of the factors previously found to be predictive of symptoms one year post-injury for this sample (age, gender, living situation, comorbidities, prior TBI, ethnicity and gender)[9] only prior TBI remained a significant predictor four-years post-injury. As additional factors such as mood and sleep quality were associated with longer cognitive symptoms this suggests that psychosocial factors play more of a role in long-term cognitive symptoms as found in other studies in Europe.[33] Identifying those with longer term risk factors and use of interventions to help with sleep quality and mood may help to improve longer-term symptom presentation following mild-TBI. The finding that baseline symptoms and injury characteristics were not significantly associated with longer-term participation outcomes suggests that other personal and contextual factors not assessed in this study may be involved and require further investigation.

Given that one third of people have been found to experience at least one TBI by the age of 25 years,[34] it is challenging and unrepresentative to recruit adult controls who have been TBI free throughout their lifetime. There is evidence that there may be cumulative effects from sustaining more than one TBI.[35] To account for the potential impact of prior TBIs on the outcome assessments, for the purposes of this study controls were included if they had experienced a TBI prior to the study timeframe. There were no significant differences between the two groups for prior TBI to control for this potential confounder. However, the timing and number of prior TBIs was based on participant self-report and is likely to be influenced by recall and diagnostic bias. Longitudinal birth cohort studies where TBIs sustained over the lifetime are more accurately recorded and verified may help to control for the impact of prior TBI on longer-term outcome.

There were higher rates of unemployment in the mild-TBI group in comparison to controls which could indicate some imbalance between the groups. The lower employment rates in the mild-TBI group are consistent with previous analyses highlighting higher unemployment rates following a mild-TBI in comparison to the NZ population.[6] Previous research has highlighted how the cognitive symptoms experienced post mild-TBI can directly impact work performance.[6, 36] Our findings support and extend this finding through mirroring the impact of cognitive symptoms on broader productivity (including both paid work as well as unpaid).

### Limitations of this study

The attrition of participants over the course of the study is a limitation which does impact the generalizability of the findings, and has been highlighted as a key source of bias in longitudinal TBI studies.[37] There were no baseline differences between the sample who consented to take part in the four year assessment and the original incidence sample. However, the findings may not be fully representative of mild-TBI as we do not know the outcomes of those who did not take part. It should also be acknowledged that these findings are based on self-reported symptoms and may be subject to reporting bias. Whilst TBI cases were directly approached about the study by the research team, controls proactively responded to advertisements in the community or were known to a research registry which may have introduced participant selection bias. Despite these limitations, the study findings have revealed evidence that mild-TBI can lead to persistent cognitive symptoms and challenges with participation up to four-years later.
